# Applications, Surface Modification and Functionalization of Nickel Nanorods

**DOI:** 10.3390/ma11010045

**Published:** 2017-12-28

**Authors:** Stefan Schrittwieser, Daniela Reichinger, Joerg Schotter

**Affiliations:** Molecular Diagnostics, AIT Austrian Institute of Technology, 1220 Vienna, Austria; reichinger.daniela@gmx.at (D.R.); joerg.schotter@ait.ac.at (J.S.)

**Keywords:** nickel, nanorod, electrodeposition, porous membrane, template synthesis, surface chemistry, functionalization, biosensing, nickel nanoparticle application

## Abstract

The growing number of nanoparticle applications in science and industry is leading to increasingly complex nanostructures that fulfill certain tasks in a specific environment. Nickel nanorods already possess promising properties due to their magnetic behavior and their elongated shape. The relevance of this kind of nanorod in a complex measurement setting can be further improved by suitable surface modification and functionalization procedures, so that customized nanostructures for a specific application become available. In this review, we focus on nickel nanorods that are synthesized by electrodeposition into porous templates, as this is the most common type of nickel nanorod fabrication method. Moreover, it is a facile synthesis approach that can be easily established in a laboratory environment. Firstly, we will discuss possible applications of nickel nanorods ranging from data storage to catalysis, biosensing and cancer treatment. Secondly, we will focus on nickel nanorod surface modification strategies, which represent a crucial step for the successful application of nanorods in all medical and biological settings. Here, the immobilization of antibodies or peptides onto the nanorod surface adds another functionality in order to yield highly promising nanostructures.

## 1. Introduction

Nickel (Ni) is a material that in the form of nanostructures is already widely employed in modern industry. Furthermore, it is also in the focus of current scientific interests. An example is in photovoltaics, where nickel oxide is used due to its semiconducting characteristics [1]. Similarly, Ni and its oxides are employed for smart windows, which control the transmission of light and solar radiation [2]. Moreover, Ni and nickel oxide also possess catalytic behavior, which, for example, is employed for the oxidation of carbon monoxide [3]. Another possible application of Ni is the field of platinum-group-metal-free hydroxide exchange membrane fuel cells. Here Ni can be employed to catalyze the hydrogen oxidation at the anode [4]. Furthermore, the ferromagnetic behavior of Ni can be employed to realize memory applications for long-term data storage [5]. Generally, the magnetic properties of nanoparticles represent an important feature that can be employed for many different applications [6,7,8,9,10]. Within this review, we focus on elongated Ni nanostructures, i.e., nanorods of cylindrical shape. Moreover, we focus on Ni nanorods that are synthesized by electrodeposition into nanoporous templates.

The underlying step for all applications involving nanorods is the nanorod fabrication itself. The different methods employed for the synthesis of metallic nanorods in general can be classified into either template-based methods (i.e., the ones focused on by this review) or template-free methods. The latter can rely, for example, on a seed-mediated growth, where metal salts are first reduced to form small nanoparticles, which act as seeds for following nanorod synthesis. To achieve elongated nanoparticle growth, structure-directing additives are employed in a second metal salt reducing step, as, for example, described by Murphy et al. [11]. Similarly, it was shown that the high-temperature decomposition of organometallic or coordination metal precursors by chemical reduction under hydrogen is a feasible method to fabricate metallic nanorods [12,13,14]. By this method, cobalt (Co) and Ni nanorods with precise geometry were prepared [15,16,17,18]. Furthermore, this method can also be employed to synthesize metallic core-shell nanorods, where a shell composed of noble metals is used to protect the inner ferromagnetic core from oxidation and, thus, degradation of the magnetic nanorod properties [19]. Another possibility for a template-free nanorod synthesis method in solution is the polyol process [20,21,22], which was employed to synthesize magnetic Co nanorod structures [23,24,25]. Recently, iron oxide nanorods have been synthesized in aqueous solution via a hydrothermal method [26] and the use of nicotinic acid as structure-directing additive, which in addition facilitates the water solubility of nanorods [27].

When specifically regarding the synthesis of Ni nanoparticles of various shapes, several different fabrication methods are reported in the literature. These include the already mentioned decomposition of organometallic precursors [12,13,17], electrochemical deposition onto flat graphite surfaces [28], chemical reduction of nickel salts in solution [29,30,31,32] and the thermal decomposition of metal-surfactant complexes under argon atmosphere [33].

The combination of the latter two paragraphs, which deal with nanorod synthesis in general and Ni nanoparticle fabrication of various shapes, is the specific fabrication of Ni nanorods. The most common synthesis method for Ni nanorods is electrodeposition into nanoporous templates. Here, the pores of a suitable template are filled with Ni to yield cylindrical nanorods within these pores. In more detail, the template is employed as electrode and immersed in a solution of Ni cations. In a next step, a voltage is applied between the template and a counter electrode so that the Ni cations are deposited inside the pores and reduced to bulk Ni. The most common employed template types are porous aluminum oxide membranes, ion track-etched polycarbonate membranes and porous silicon [34,35,36]. This synthesis method was established in the mid-1990s by Martin and Al-Mawlawi et al. [37,38,39] and is in the meantime well documented in the literature by various reviews [36,40,41,42,43]. Generally, a huge variety of different materials has been explored with regard to the synthesis of nanorods by electrodeposition into porous templates. In a recent review, Davydov and Volgin report on template-assisted metal electrodeposition and list metals that have been communicated in this context [42]. This includes next to the ferromagnetic materials iron, nickel and cobalt, also gold, platinum, silver, tin, tellurium, lead, silicon and many more, including various alloys and chemical compounds. Other examples of materials employed for deposition into porous templates are perovskite [44], palladium [45], rhodium [46] and carbon atoms arranged as fullerenes [47]. Next to the synthesis of nanorods consisting of a single metal, segmented nanorods composed of multiple layers of different metals have also been reported [48,49,50,51]. Thus, the electrodeposition technique represents a very versatile method to fabricate a wide range of different nanostructures.

Once the nanostructures are fabricated, they have to be released from the template first before they can be stabilized in solution. A suitable surface modification allows provision of them with a specific functionality. This is especially true for medical and biological applications, where the nanoparticles need to be stabilized in a complex biological medium and, moreover, need to fulfill a certain medical or sensing task. Different methods to modify and functionalize the surface of nanoparticles of various composition and size are documented in the literature [52,53,54,55,56,57]. Within the current review, we will discuss surface modification approaches that are reported for electrodeposited Ni nanorods.

In the following sections, we will, first, discuss the applications of nickel nanorods and then move on to strategies for surface modification and functionalization. Finally, we conclude with an outlook in which we also propose alternative possibilities for nanorod surface modifications.

## 2. Nickel Nanorod Applications

This section discusses applications of electrochemically synthesized Ni nanorods that have been reported in the literature. An already widely discussed application is so called micro- and nanomotors, which are self-propelled by locally provided chemical fuels to drive these devices in solution. They often rely on particles with a Ni content, which is used to guide the particles through a sample solution by applying external magnetic fields. Numerous examples for differently surface-functionalized nanomotors that fulfill certain application-dependent tasks can be found in the literature [58,59,60,61,62].

For all possible applications, it is of fundamental importance to have a suitable Ni nanorod characterization. This allows understanding of the physical behavior of nanorods in various settings as well as the results gained by their application. Consequently, many studies in the corresponding literature deal with the different properties of Ni nanorods that are synthesized by electrodeposition into porous templates. Among others, the studied topics account for optical, magnetic, crystallographic and compositional properties [63,64,65,66,67,68,69,70,71,72,73,74,75,76,77,78,79,80,81,82,83,84,85,86,87,88]. Within the current review, we focus on Ni nanorod applications that enable to examine phenomena that are additional to the determination of the physical properties of bare Ni nanorods only. The various fields of Ni nanorod applications are discussed in the chapters below. A short summary of these is given in Table 1.

### 2.1. Rheological Fluid Properties

The first to employ magnetic particles to determine rheological fluid properties were Crick and Hughes in 1950, who examined the elastic properties of cytoplasm in chick fibroblasts [145,146]. To that end, they employed cell cultures, magnetic particles and external magnetic fields [145,146]. Protocols that have been established in this work are still of interest today, as recently demonstrated by the group of Andrejs Cebers, who applied iron oxide nanorods for microrheological measurements of gels of the bacteriophage Pf1 [147].

**Figure 1:**
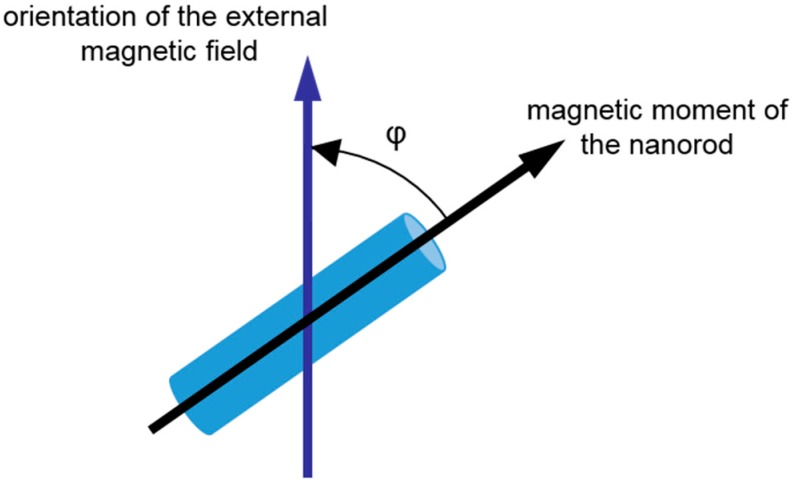
Sketch of a magnetic nanorod and an external magnetic field. The angle between the orientation of the magnetic field and the direction of the magnetic moment of the nanorod is denoted as ϕ.

Generally, in order to examine rheological fluid parameters, magnetic nanorods are immersed in a sample solution and their movement under application of an external magnetic field is studied. A schematic illustration of a magnetic nanorod in an external magnetic field is shown in Figure 1. The angle ϕ describes the orientation of the magnetic moment of the nanorod to the direction of the external magnetic field. When being dispersed in solution or embedded in a soft matter, the nanorod will rotate and orientate parallel to the external field. This rotation depends on the physical properties of the nanorod (i.e., geometry and magnetic moment), the strength of the external magnetic field and the rheological fluid parameters. Thus, the latter can be deduced by observations of the nanorod behavior in solution. This can be accomplished by magnetic or microscopic measurements.

Ni nanorods have been employed as local probes to examine the swelling behavior of chemically cross-linked poly(acrylamide) hydrogels by a quasi-static magnetometry approach using a vibrating sample magnetometer device [89]. The results show that the measured coercivity depends on the concentration of the applied chemical cross-linker and, thus, on the shear modulus of the hydrogel matrix. Similarly, the shear modulus of gelatin gels has been determined in dependence of the gelatin concentration [90]. Estimates for the lower and upper bound of the shear modulus were made possible by the distribution of the Ni nanorod geometry parameters. This procedure was then further developed to test soft and hard matrix properties (depending on the employed gelatin concentration) and to determine the local shear modulus of the gel. To that end, an external magnetic field was applied during the gelation process to align the embedded nanorods, which made it possible to measure hysteresis curves parallel and perpendicular to the orientation of the magnetic nanoparticles [91]. For the case of a soft matrix and magnetization measurements perpendicular to the nanorod orientation, the measured susceptibility value could only be explained by an additional rotation of the nanorods. This did not occur for hard matrix properties. Tokarev et al. studied the time-dependent microrheology of a hydrogel during the photopolymerization process by applying external magnetic fields to Ni nanorods immersed in the hydrogel solution and optical observation of their rotational dynamics [92,93]. The same group applied their measurement method to study the rheological properties of butterfly saliva in a volume of a few nanoliters [148].

Another possibility for an optical observation of Ni nanorods immersed in a sample solution to study its microrheological properties was presented by Tschöpe et al. [94]. They developed a procedure based on the magneto-optical response of the employed Ni nanorods. Specifically, they measured the optical transmission of a sample solution containing the Ni nanorod probes excited by external rotating magnetic fields with incident linearly polarized light. Here, the rotation of the nanorods in solution depends on their hydrodynamic volume and the rheological fluid parameters, which can be deduced by measurements of the rotational nanorod behavior. By this method, the transition of a gelatin sol from a viscous fluid to a viscoelastic hydrogel and the relaxation of a CTAC/NaSal (cetyltrimethyl ammonium chloride and sodium salicylate) wormlike micellar solution were examined. The microrheology of a wormlike micelle solution of cetylpyridinium chloride–sodium salicylate was also studied by analyzing the torque of nanorods excited by external magnetic fields, which was achieved by microscopic observations of the nanorod orientation with respect to the applied magnetic field [95].

Ni nanorods have also been employed to characterize interfacial shear rheology. Specifically, Ni nanorods were applied to study the interface of a water and glycerol mixture with a polyphenyl-methylsiloxane silicone oil film on its top surface by microscopic observations of the nanorod orientation in external magnetic fields [96]. A further example of studying interfacial rheology parameters with Ni nanorods is reported by Dhar et al., who examined the viscosity of albumin at an air-water interface and its change over time [97]. The authors employed the detection of the orientation of Ni nanorods in externally applied magnetic fields. During ageing of the protein film, the surface viscosity increased by 4 orders of magnitude within the first two hours, which proves the wide dynamic range of the measurement method.

### 2.2. Data Storage, Electronics, Catalysis and Optical Phenomena

The magnetic properties of Ni nanorods make it obvious to apply this kind of nanostructure to magnetic data storage applications. In current magnetic data storage devices the magnetic domains on a bulk surface of a magnetic material form a single bit. This can also be realized by single nanorods or single segments of segmented nanorods. To that end, multilayered nanorods composed of alternating Ni and Cu segments were compared to bulk nickel with regard to their coercivity and saturation magnetization values [98]. It was concluded that these segmented structures can be applied for high-density magnetic storage devices showing a bit density of up to 70 Gbits/in^2^. Even higher bit densities of ~3 Tbit/in^2^ were achieved by electrodeposited Co nanorods, which prove the potential of electrodeposited magnetic nanorods for data storage applications [149].

Microwave electronics presents a further possible field of application for electrodeposited Ni nanorods. Encinas-Oropesa et al. suggested to use nanorods deposited inside the pores of a highly ordered membrane to be applied for wide band tunable electric filters [99]. This statement is supported by measurements of the ferromagnetic resonance properties, which occur even at zero magnetic field, in a frequency range of 100 MHz to 40 GHz, and it was shown that the obtained ferromagnetic resonance properties depend on the nanorod density inside the membrane, which is a tunable parameter. Similar measurements are also reported by Ramos et al. and Kuanr et al. [100,101]. The latter publication also compares measured to calculated resonance curves, and, similarly to the above mentioned article of Encinas-Oropesa et al., the authors also determine a zero field resonance [101]. Microwave devices such as circulators or filters for wireless communication and automotive systems are also described in literature. Specifically, the design of a planar circulator operating at zero external DC bias field is discussed [102]. The ferromagnetic resonance behavior of a planar inductor on a substrate, i.e., Ni nanorods in an alumina membrane, was studied by Hamoir et al. [103]. They could show that the usage of high aspect ratio nanorods results in an increase of the inductance as well as of the quality factor, both of which depending on the membrane pore density and the height of the filling. Ni nanorods were also employed as microwave filter devices once they were released from the substrate by chemical etching processes, which was shown in a recent publication [104]. Here, nanorods were dispersed and aligned in a silicone matrix to study their frequency response to an externally applied microwave signal.

**Figure 2:**
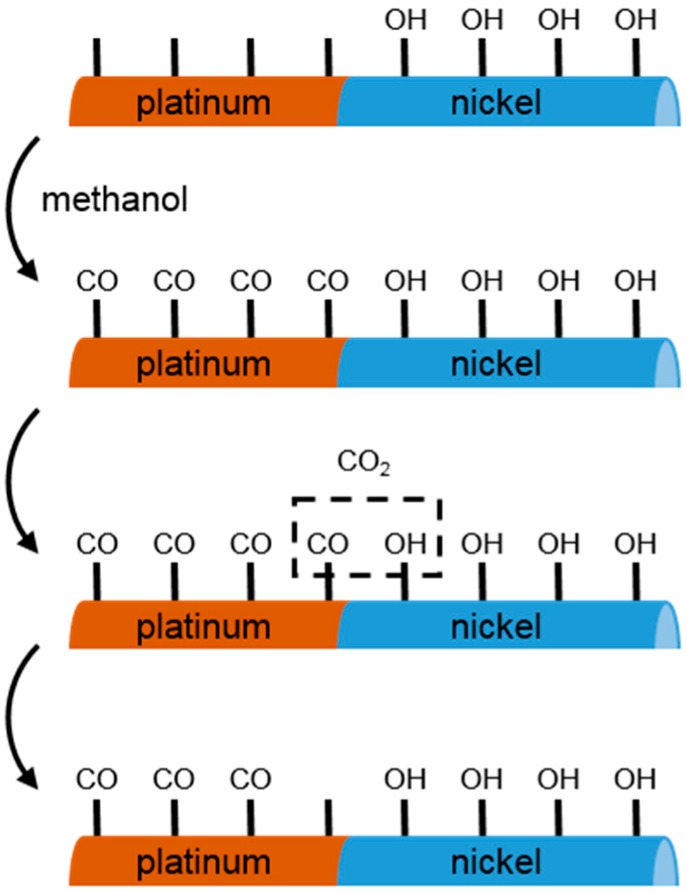
Illustration of the platinum reactivation process by nickel for the catalysis of methanol. The surface of the native oxide on top of the nickel segment is covered by hydrogen oxide groups (OH) in an aqueous dispersion. In the presence of methanol, the reaction sites on the platinum segment will be occupied by carbon monoxide species (CO). These CO groups react with the neighboring OH groups to form carbon dioxide (CO_2_). The result is that the reaction site on the platinum is available for the next reaction. Adapted figure from [51] with permission from copyright (2004) American Chemical Society (Washington, DC, USA).

Next to applications in electronics, Ni nanorods in combination with platinum are also employed for catalysis applications. This can be exploited for methanol fuel cells, as it was demonstrated for nanorods composed of nickel and platinum segments [51,105]. Here, a common problem is the poisoning of the platinum by carbon monoxide-like species. This can be improved by a nickel-platinum bimetallic structure, where the hydroxy species on the nickel in aqueous solution transform the carbon monoxide-like species to carbon dioxide, thus reactivating the platinum (see Figure 2 for a schematic sketch) [51,105]. Recently, enhanced electrocatalytic oxygen reduction characteristics were reported for the same kind of nanorods in comparison to spherical nanoparticles [106].

Regarding the use of Ni nanorods for studying optical phenomena, it was reported that Ni nanorods can be employed to examine plasmon resonance excitations in two component nanorods composed of alternating Ni and Au segments [107]. It was shown that the free electrons in Ni also contribute to the plasmon excitations and that these free electrons control the optical coupling between the Au segments. This can be potentially further employed for the design of plasmonic waveguide structures. Similarly, plasmonic properties and optical coupling were examined for Au segments that are fixed on a substrate [108]. Separated Au segments were achieved with segmented Ni-Au nanorods after wet-chemical removal of the Ni parts once the nanorods were adhered to the substrate. Recently, Jung et al. examined localized surface plasmon resonances (longitudinal and transversal) of segmented Ni-Au nanorods in aqueous solution [109]. Specifically, they applied external magnetic fields aligning nanorods in solution to study their absorption behavior in dependence of the direction of light polarization and in dependence of the number of Ni and Au segments and their corresponding lengths.

Another example of an optical phenomenon that was studied by using electrodeposited nanorods is surface-enhanced Raman scattering (SERS). This was shown by nanorod arrays (Co, Ni, Pt, and Pd nanorods) freestanding on a conductive electrode, for which the optical properties were analyzed experimentally and theoretically under consideration of the nanorod material and geometry [110]. These findings have been confirmed by similar ones obtained by Sauer et al., who applied Raman microscopy to Ni nanorods [111]. Also, nanorods consisting of Ni-Au segments were employed to prove a long-range SERS effect when the Ni segments only were modified with a Raman-active probe and locally separated from the Au segments [112]. The separation distance was up to 120 nm, which avoids coupling of the excited plasmon resonances. This long-distance effect enables to determine the actually active Raman segment by confocal microscopy and allows for a reduction of quenching effects when fluorophores are employed.

Finally, dispersions of Ni nanorods were employed to study their optical transmission using incident linearly polarized light in dependence of an external magnetic field [113,150]. The so obtained results suggest the use of Ni nanorods for optical switches that are controlled by magnetic fields, which can be applied for liquid crystal technology [113]. Similarly, cold cathode fluorescent lamps used for optical applications can be realized by using Ni nanorods as reported by Feizi et al. [114]. These are of interest for liquid crystal displays. To that end, Feizi et al. demonstrated an enlarged electron emission by a factor of four at a bias potential of 1 V in comparison to bulk nickel substrates.

### 2.3. Sensing and Biosensing Applications

Nickel nanoparticles synthesized in porous membranes can be applied for the sensing of carbohydrates. To that end, Lu et al. synthesized nickel nanorods in polycarbonate membranes that were placed on a glassy carbon electrode to end up with a freestanding nanorod array on the electrode after chemically dissolving the polycarbonate template [115]. This electrode served as working electrode for non-enzymatic electrochemical detection of glucose by cyclic voltammetric and amperometric measurements. A detection limit of 100 nM was achieved in an alkaline solution. Carbohydrates were also detected in a non-enzymatic electrochemical detection by first dropping a solution of dispersed nickel nanorods onto the surface of a screen printed electrode [116]. Then, the Ni nanorods were activated by an amperometry step in alkaline solution to generate oxygen groups on the surface, which were employed for catalytic oxidation of carbohydrates. A detection limit of 20 µM for glucose was achieved in serum samples, which typically show glucose concentrations in the lower millimolar regime. The same measurement method was also applied for the sensing of inulin in buffer solutions [117]. Recently, this measurement method was further developed into a miniaturized flow injection analysis system to detect galactose in urine of newborns for the diagnosis of galactosemia, a genetic disorder impairing galactose metabolization [118]. Nickel nanorods coated by electroless deposition with a gold layer freestanding on a substrate were used as electrode for the detection of glucose [119]. The actual sensing was carried out by cyclic voltammetry measurements. Here, nanorods were employed to increase the electrode surface, while the detection of glucose was achieved via the enzyme glucose oxidase. A non-enzymatic approach for the detection of glucose with the help of a nanorod array was recently presented by Qin et al. [120]. To that end, they synthesized multilayered nickel gold nanorods on the surface of an electrode by using an alumina membrane that was chemically dissolved after nanorod synthesis. Amperometric measurements were conducted to detect glucose in sodium hydroxide solutions with a detection limit of 0.1 µM and, furthermore, glucose was also detected in spiked urine samples.

**Figure 3:**
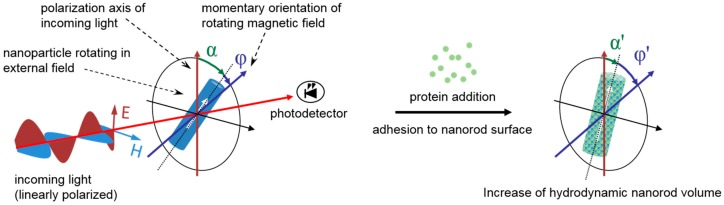
Measurement principle for detecting proteins in solution according to reference [121]. Nanorods immersed in solution were excited by an external rotating magnetic field, which caused the nanorods to rotate with field, but delayed by a phase lag ϕ. This is caused by the drag torque that the nanorods experienced in the solution. The phase lag ϕ was a function of the hydrodynamic nanorod volume. The orientation of nanorods immersed in solution was measured optically by linearly polarized light. By measurements in transmission geometry, the detected intensity depended on the angle α between the direction of light polarization and the long axis of the nanorod. An increase of the hydrodynamic nanorod volume was observed upon addition of a protein to the sample solution, which leads to protein adhesion to the nanorod surface. This resulted in a change of the angle ϕ and, thus, of the angle α, thereby leading to a measurable change in the optical signal. Adapted figure from [121] reprinted with permission from copyright 2014, John Wiley and Sons (Hoboken, NJ, USA).

Next to the detection of carbohydrates, Ni nanorods in an aqueous dispersion can also be employed for the detection of proteins in solution. This was shown for the bovine serum albumin protein, which adsorbed to the surface of Ni nanorods [121]. The measurement principle was based on a change of the hydrodynamic volume due to protein adsorption and was measured optically under excitation of the nanorods by an external rotating magnetic field [151]. By using linearly polarized light, the momentary orientation of the nanorods in solution can be obtained employing measurements in transmission geometry [121]. This was due to the anisotropic optical scattering behavior of elongated nanostructures (see Figure 3 for details on the measurement method). While the adsorption of bovine serum albumin is an unspecific reaction of the protein with the nanorod surface, specific detection of target proteins was shown for antibody functionalized nanorods of a different type, i.e., Co core nanorods covered by a noble metal shell [152,153]. Thus, it can be deduced that similar measurements can be made possible for Ni nanorods with a suitable surface functionalization.

The magnetic properties of Ni nanorods already suggest that they can also be employed for magnetic resonance imaging applications. Recently, Bañobre-López et al. presented the application of Ni nanorods as contrast agents for magnetic resonance imaging [122]. To that end, stable dispersions of Ni nanorods with a surface coating of polyacrylic acid were prepared, and their transverse relaxivity r2 was determined. It was concluded that these nanorods possess relaxivities comparable to commercially available T2 contrast agents and, thus, are well suited in magnetic resonance imaging applications.

Alternatively, the magnetic properties of Ni nanorods can be applied for magnetic separation techniques. To that end, a sensing method was presented by Pinheiro et al. for the specific detection and targeting of mercury ions [123]. Here, the heavy metal ions bound to the nanorod surface so that they could be removed from a solution by magnetic separation. In order to target heavy metal ions, the surface of the Ni nanorods was modified to finally achieve silica shell coated nanorods with incorporated dithiocarbamate groups that show a strong affinity for binding heavy metal ions. The feasibility of this method was shown by applying the nanorods to remove mercury from an aqueous solution by magnetic separation with a removal rate of 99.8% of mercury ions. The resulting final mercury concentration value was below the recommended guideline threshold for drinking water quality in Europe.

### 2.4. Cell Biology Applications

The first reported applications of magnetic particles for cell biology dealt with the characterization of the rheological parameters of the cytoplasm in chick fibroblasts as detailed above in Section 2.1. In recent years a growing number of reports can be found that focus on the cellular response once nanorods were internalized by cells and then agitated externally by magnetic fields. For example, Castillo et al. studied the torques of segmented Ni-Pt nanorods when incubated with fibroblast cells under excitation by an external magnetic field [124]. The nanorod rotation was observed microscopically and as a result, they were able to define a minimum torque for nanorod rotation for which they deduced a non-Newtonian behavior of cytoplasm. A further example for a cellular response to external force was investigated by pulmonary artery smooth muscle cells with internalized Ni nanorods and an array of flexible micropost force sensors as a substrate for cell adhesion [125]. External magnetic fields of low frequency up to 10 Hz were applied, and the cellular contractile force was studied. Furthermore, rotational motion of nanorods internalized by different cells was used to study the effect of gene mutations as demonstrated by Celedon et al. using Lamin A/C knockout cells that showed defective nuclear mechanics [126]. To that end, the nanorods were incubated with the cells so that they got internalized, and their rotational motion excited by an external magnetic field was studied optically to deduce viscosity and elasticity parameters of the cell nucleus.

**Figure 4:**
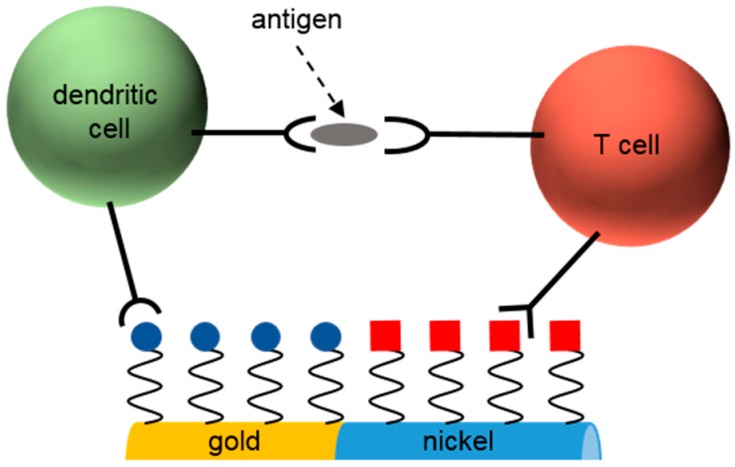
A Ni-Au segmented nanorod acting as bridge between a dendritic cell and a T cell. Both cell types were bound to the nanorod via specific surface functionalization. This resulted in a close vicinity of the dendritic and the T cell. As a result an antigen was presented to the receptor of the T cell. Adapted figure from [132] reprinted with permission from copyright (2013) American Chemical Society.

Ni nanorods can also be employed to release a specific molecule inside cells or to present a chosen molecule to cells. The aim of both approaches is to trigger a cellular response. For this purpose, specially designed segmented nanorods composed of Au, Ni and polypyrrole were applied for a controlled release of adenosine triphosphate molecules bound to the polypyrrole under application of a negative electrical potential [127]. This proved the use of these specially designed nanorods for the storage and the controlled release of chemical species that were initially immobilized onto the nanorod surface. To that end, the nanorods were connected on both ends by an electrode, where the Ni segment is employed to facilitate oriented electrical connection by externally applied magnetic fields (nanorod orientation perpendicular to the gap between two electrodes). Another example for a controlled release of target molecules was given by Salem et al., who demonstrated the use of segmented Ni nanorods to release a small DNA molecule inside a targeted cell [128]. Specifically, they bound the DNA to the Ni segment with a molecule providing a disulfide bond that was cleaved by the chemically reducing conditions inside the cell. Incorporation of the nanorods into cells was facilitated by the binding of transferrin proteins as ligands for receptor mediated endocytosis to the gold segment of the nanorods. This method can be employed for gene therapy that aims to incorporate DNA into cells to trigger a defined biological function. Similarly, Choi et al. functionalized Ni-Au segmented nanorods by small interfering RNA to knock down the vascular endothelial growth factor protein, and by luteinizing hormone-releasing hormone peptides so that the nanorods can target cancer cells [129]. These segmented nanorods were incubated with a breast cancer cell line, and it was observed that the nanorods specifically target cancer cells where the small interfering RNA as the anticancer agent caused cell apoptosis. Moreover, segmented Ni-Au nanorods were also applied for the delivery of the chemotherapeutic drug doxorubicin into the cytoplasm of cells. This was achieved by immobilizing the drug onto the surface of the nanorods together with a cell specific ligand to facilitate the endocytosis of the nanorods [130]. While the latter examples demonstrate the utility of nanorods to release a specific target molecule, Ni nanorods were also employed to trigger a cellular response when simply brought in contact with the cells. In order to achieve this, the nanorod surface has to be functionalized with a bioactive molecule. This was shown by Sharma et al., who applied Ni nanorods with Au caps on both ends with a peptide sequence of three amino acids bound to the particles’ surface [131]. This peptide sequence interacted with the integrin transmembrane receptors, a protein in the cell membrane responsible for generating cellular signals that regulate the cell cycle. With the help of the specific surface functionalization and comparison to nanorods with a polyethylene glycol surface, it was possible to increase the nanorod dispersal and also the cell density, indicating a good cell viability. Finally, a cellular reaction to nanorods can also be achieved by nanorods comprising two individual surface functionalities. By Ni-Au segmented nanorods with specific surface functionalization on each metal segment, it was shown that the nanorods can serve as a bridge between dendritic cells and T cells, which results in an immune response and the release of cytokines [132]. As a consequence, the nanorods can be employed to study the antigen presentation process to the T cells when incubated together with the cell lines of interest (see Figure 4 for a schematic sketch).

A further possibility to apply Ni nanorods together with cells is to guide the entire cell to a specific location or to guide the cell growth along a specific direction. This can be achieved by making use of the magnetic properties of nanorods and external magnetic fields to manipulate the orientation of nanorods. For example, cells that internalized Ni nanorods were guided to the gap between a ferromagnetic and a gold electrode by magnetic interaction of the electrode with the Ni nanorods to allow for non-destructive examination of neurons, i.e., a cell type responding to electrical signals [133]. A further example was demonstrated by Tanase et al. who controlled the organization of mammalian cells using Ni nanorods in conjunction with a patterned micromagnetic substrate [134]. Cells could be adsorbed to the nanorod surface, which did not hinder the external orientation of the nanorods by the micromagnets and by an additional external magnetic field so that controlled organization of two dimensional cell structures was achieved. An alternative method for cell guidance was presented by Johansson et al. who employed Ni nanorods that were dispersed on a substrate and oriented by an external magnetic field to form structures for subsequent cell adhesion [135]. Here, not only the employed fibroblast but also the axons showed oriented adhesion along the main nanorod axis. Another example of lateral cell displacement by the effect of nanorod rotational motion caused by external magnetic fields was demonstrated by using Ni nanorods and skeletal myoblasts, which can be employed to create three dimensional cell clusters [136].

Comparable to the guidance of the cells as described in the paragraph above, Ni nanorods can also be employed to execute cell separation. Hultgren et al. demonstrated the application of Ni nanorods for cell separation by an external magnetic field after incubation of the employed cells with the magnetic nanorods [137]. The same research group further studied the influence of the nanorod length on the cell separation performance and concluded that the cell separation performance is best when the nanorod length is comparable to the cell diameter [138]. Similarly, Nanorod dispersions with two different nanorod lengths were employed to capture cells of a specific diameter in accordance to the nanorod length and cell diameter correlation [139]. More recently, a cell separation technique was presented by Gao et al. who applied nanorods with an antibody surface functionalization for a proof of principle of cell separation in an external static magnetic field [140]. Briefly, the authors incubated cells and Ni nanorods and placed the mixture in a static magnetic gradient field to separate cells with internalized nanorods from nanorod-free cells by removing the supernatant and, finally, counted the number of separated cells by cytometry.

Inducing a controlled cell death is an important application of magnetic nanoparticles that is employed for cancer therapy [154]. In that regard, Fung et al. demonstrated the application of Ni nanorods internalized by fibroblasts to induce an inflammatory response by the cell and subsequent cell death [141]. This was achieved by external magnetic fields at a low frequency of 1 Hz to induce nanorod motion, which caused upregulation of the IL-6 cytokine and cell death. An experimental proof was given by colorimetric cell viability assays. Similar results with a similar nanorod excitation were obtained for human embryonic kidney cells [142]. Additionally, induced cell death of cancer cells with internalized Ni nanorods by manipulation of nanorod motion with external AC magnetic fields was demonstrated by Contreras et al. [143]. Here, a weak magnetic field with an amplitude of 0.5 mT only was applied and it was shown that this field strength is enough to induce mechanical cell disturbances that result in reduced cell viability. This was explained by cell membrane ruptures caused by the nanorods and their motion due to the external excitation. Recently, Hopkins et al. presented a method for tumor therapy based on a radio frequency induction of heat in Ni nanorods covered by a Au shell [144]. Here, Ni nanorods were fabricated by electrodeposition into alumina membranes followed by a Au shell synthesis achieved via electroless plating of the Ni cores. These particles were injected into xenograft mice models. Specifically, the tumor cells of a pancreatic tumor were transplanted into the animals to grow the tumor into which the nanorods were injected. The radio frequency signal was generated by an external microstrip spiral antenna and the effect of heat induction was based on eddy currents on the nanorod surface and a loss of hysteresis due to the applied AC field. These resulted in a nanorod heating of up to 45 °C that causes severe damage to the neighboring tumor cells.

An important topic for all medical applications involving nanostructures of any geometry is the cytotoxicity of the applied nanostructures. The cytotoxicity of Ni nanorods depends on many factors, as for example the geometry, the surface coating, but also the measurement procedure for evaluation of cytotoxic effects. Consequently, while some studies can be found in literature on the cytotoxic effects of Ni nanorods, they can hardly be compared [155,156,157]. A reasonable examination of the cytotoxic effects of Ni nanorods is in our opinion best conducted for the specific type of nanorods and under consideration of the envisaged application. Thus, a deeper discussion of this topic is omitted within this review.

## 3. Nickel Nanorod Surface Chemistry Modification

Chemical modification of the surface of Ni nanorods is essential for a lot of complex applications as they have been detailed in the previous section. A prerequisite for applying nanorods is to separate them from the nanoporous template in which they were synthesized. The two most common template materials for electrodeposition are aluminum oxide and polycarbonate, which have to be removed without damaging the Ni nanorods in the pores. This can be achieved by chemically selective etching processes. While the polycarbonate membrane can be rapidly dissolved in dichloromethane, the aluminum oxide template can be dissolved in sodium hydroxide solutions or in mixtures of chromic acid and phosphoric acid [42]. Though functional groups can be immobilized onto the nanorod surface by an unspecific binding process (e.g., as demonstrated for binding of streptavidin protein by an incubation step in a cell culture medium [133]), more specific binding processes are addressed by most research groups. Another exception for an unspecific binding process to the nanorod surface was presented by Sharma et al., who modified the negatively charged surface of Ni nanorods by positively charged amine groups via ionic binding processes [131]. This was controlled by the adjustment of the pH value of the dispersion solution under consideration of the isoelectric point of the employed molecules. Similarly, Magnin et al. employed the layer-by-layer technique [158], which is based on the coating of the surface by multiple polymer layers of opposite charge [159]. Specifically, positively charged chitosan and negatively charged carboxymethylpullulan layers were immobilized onto the nanorod surface. In the following subsections, different types of chemical surface modifications that are structured with respect to the employed molecule and the anchor group for linkage to the nanorod surface will be discussed. An overview is given in Table 2. For this report, we only considered references with a detailed description of the experimental procedure so that it can be reproduced by others.

Commonly and as mentioned above, the surface modification of the synthesized nanorods is employed after removal of the nanoporous template, but it is also possible to modify the walls of the pore channels before electrodeposition to yield nanorods with a specific surface functional group. An example for a surface modification inside the pore channels before electrodeposition was presented by Skinner et al., who synthesized segmented Au-CdSe nanorods with exclusive modification of the Au sections [172]. Specifically, they employed vapor deposition to cover the walls of the pore channels by (3-mercaptopropyl)trimethoxysilane (MPTMS), a molecule comprising a silane group and a thiol group separated by a short carbon chain. Modification of the nanoporous template was executed before each Au deposition step through the silane group that reacted with the aluminum oxide, and once the gold was deposited, the thiol groups attached to the gold surface. A cleaning step to remove the MPTMS molecules from the surface was executed by an oxygen plasma etching process before electrodepositing CdSe. Channel wall surface modification prior to the electrodeposition was also presented by Sanz et al. for Ni nanorods, which were coated by a polystyrene layer inside the pores [173]. This was achieved via formation of polystyrene nanotubes in the pores of an alumina template by placing a polystyrene film on top of the template, followed by an incubation at 200 °C in a nitrogen atmosphere, which resulted in the adhesion of a polymer film on the pore walls. Next, the polystyrene nanotubes were filled with Ni in an electrodeposition step, and the alumina template was removed in a sodium hydroxide solution so that, finally, Ni nanorods in a polystyrene shell were obtained in solution.

### 3.1. Small Molecules Based on Carboxylic Acid Groups

**Figure 5:**
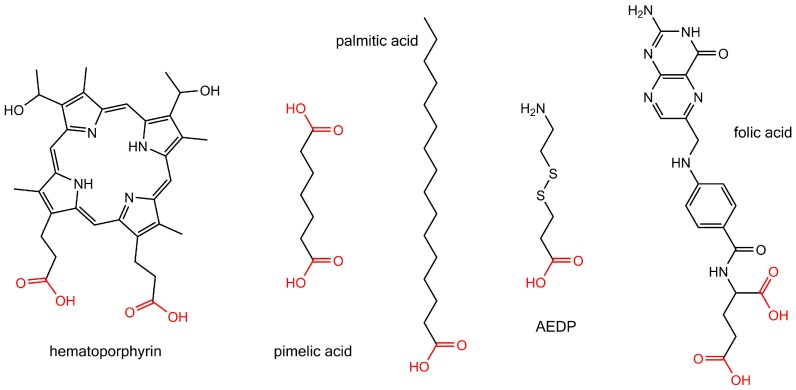
Molecules for Ni nanorod surface modification based on carboxyl groups. The carboxyl groups are shown in red. From left to right these molecules are: hematoporphyrin, pimelic acid, palmitic acid, 3-[(2-aminoethyl)dithio] propionic acid (AEDP), and folic acid.

A widely employed method for Ni nanorod surface modification is based on carboxylic acids, which have a high affinity to the native nickel oxide layer that is present on top of the bare nickel nanorod [174,175]. The binding process of a carboxylic acid to a metal surface may involve the generation of metal-carboxylate salts or adhesion of the carboxyl group to the metal, which can be accompanied by a proton transfer from the carboxyl group to an oxygen atom on the metal surface, which in the case of Ni nanorods stems from the native oxide layer [175]. The here-discussed molecules carrying a carboxyl group for Ni nanorod surface modification are depicted in Figure 5.

An early report on the surface modification of electrodeposited Ni nanorods by employing carboxyl groups for surface linkage was presented by Tanase et al. [160]. Here, the authors modified the Ni nanorod surface by Hematoporphyrin IX, a fluorescent porphyrin that possesses two carboxyl groups for the binding process. The modification of the nanorod surface was achieved after dissolving the alumina template in a solution of potassium hydroxide to remove the nanorods from the template. This was followed by a washing and transfer step of the nanorods in solution to ethanol by centrifugation and magnetic separation using permanent magnets. The binding of Hematoporphyrin IX was executed by mixing the porphyrin with the nanorods and immersion for 24 h at room temperature followed by another solvent washing step with ethanol to remove unbound porphyrin species. This procedure was expanded to also transfer the nanorods in solvents of water and ethylene glycol [161].

Gao et al. presented a method to functionalize the nanorod surface by biotinylated antibodies via modification of the nickel surface with carboxyl groups, followed by binding of a streptavidin protein layer, which has a very high affinity to the biotin groups of the antibody [140]. The reported protocol involved the use of pimelic acid, a dicarboxylic acid molecule that serves for both the binding to the nickel nanorod surface and as anchor for amine groups of the streptavidin. The latter is subsequently bound to these anchors via EDC/S-NHS linker chemistry (EDC: *N*-(3-(Dimethylamino)propyl)-*N*′-ethylcarbodiimide hydrochloride; S-NHS: *N*-Hydroxysulfosuccinimide sodium salt). In a first step, the alumina template was dissolved in a sodium hydroxide solution, and the nanorods were transferred into ethanol by centrifugation. Then, the nanorods were immersed in ethanol containing pimelic acid and incubated for 24 h. The EDC/S-NHS coupling chemistry was conducted by adding phosphate buffered saline (PBS) solutions of EDC and S-NHS together with the streptavidin protein, followed by incubation for 3 h. Finally, the biotinylated antibody was immobilized by incubation with the streptavidin-coated nanorods for 30 min in PBS solution. Washing and solvent transfer steps were conducted after each individual surface modification step.

Several examples for surface modification of segmented Ni-Au nanorods can be found in literature, all of which employ thiol groups for modifying the gold segments and carboxyl groups for nickel surface modification [128,130,132,162]. Birenbaum et al. employed palmitic acid for modifying the surface of the Ni segments to yield hydrophobic Ni segments (due to the alkyl group of palmitic acid) [162]. The palmitic acid was immobilized on the nanorods by immersion in a solution of ethanol overnight. Salem et al. presented functionalization of nickel segments by 3-[(2-aminoethyl)dithio] propionic acid (AEDP), which is a molecule providing a carboxyl and an amine group spaced by a short alkyl segment and a disulfide bridge [128]. Here, the binding was achieved in a solution of AEDP in water after 24 h. At a pH value of 5.7, small plasmid molecules (circular double-stranded DNA) were bound by electrostatic interaction to the positively charged amine groups of the AEDP in a further incubation step that lasted for 24 h. It was also reported to bind folic acid to the Ni segments of a bimetallic nanorod via the carboxyl groups of folic acid by immersion in methanol at 4 °C for 12 h [130].

### 3.2. Small Molecules Based on Silane Groups

**Figure 6:**
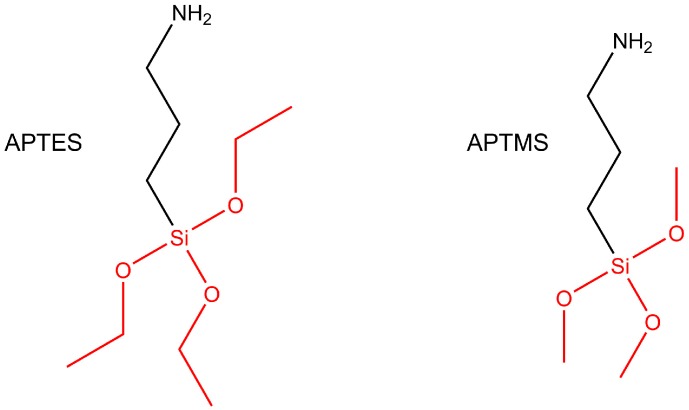
Molecules for Ni nanorod surface modification based on silane groups. The silane groups are shown in red. From left to right, these molecules are: (3-aminopropyl)triethoxysilane (APTES) and (3-aminopropyl) trimethoxysilane (APTMS).

Silanization of a metal oxide surface is a well-established process that is based on a molecule containing a silane group and a functional end group, which are separated by an alkyl chain [176,177]. Silane groups are based on a silicon atom linked to other atoms with single bonds in a tetrahedral geometry, with the silicone atom in the center. The binding to the metal oxide substrate can rely on the hydration of the latter, which means that hydroxyl groups are present on the surface [176,177]. In a self-assembly process, the silane head groups form covalent bonds with the hydroxyl groups. The here-discussed molecules carrying a silane group for Ni nanorod surface modification are shown in Figure 6.

Wildt et al. presented surface modification of Ni in segmented Ni-Au nanorods by using a silane group for the coupling reaction to the native oxide on the nickel segment [163]. Here, the authors first dissolved the alumina template in a solution of potassium hydroxide, followed by washing and solvent transfer steps to yield nanorod solutions in ethanol. Coupling of a silane group to the nanorod surface was executed by immersion of the nanorods in ethanol with (3-aminopropyl)triethoxysilane overnight. This resulted in a nanorod surface that is now terminated by amine groups that can be further employed to do a subsequent surface modification step, e.g., by *N*-hydroxysuccinimide esters. Wildt et al. employed a methoxypoly(ethylene glycol) succinate *N*-hydroxysuccinimide ester to bind polyethylene glycol to the nanorod surface, which was done by immersion for 1 hour in a solution of sodium bicarbonate.

Recently, Kozlovskiy et al. demonstrated the surface modification of Ni nanotubes by (3-aminopropyl) trimethoxysilane, which was achieved by mixing the nanotubes with the silane reagent in ethanol [164]. The so obtained solution was incubated for 12 h after an initial sonication step. The resultant amine terminated surface was further modified to bind bovine serum albumin (BSA) protein via its carboxyl groups. The protein linking was done in an acetate buffer solution of acidic pH and under addition of *N*-(3-(Dimethylamino)propyl)-*N*′-ethylcarbodiimide and pentafluorophenol to activate carboxyl groups of BSA, thus making them bind to the amine groups on the nanorods.

### 3.3. Surface Modification by Polymers

**Figure 7:**
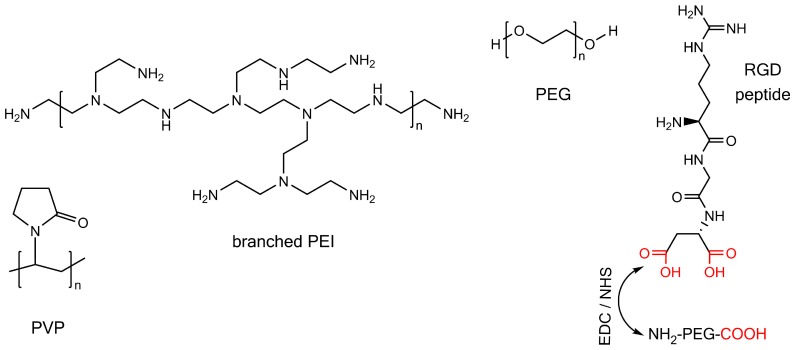
Polymers for Ni nanorod surface modification. From left to right, these polymers are: polyvinylpyrrolidone (PVP), branched polyethyleneimine (PEI), polyethylene glycol (PEG), and a RGD peptide bound to a polyethylene glycol modified with a carboxyl and an amine group. Carboxyl groups are shown in red. The amine group of the PEG is bound to the RGD peptide via EDC/NHS linker chemistry.

The use of polymers for nanoparticle surface coating offers some distinct advantages. Very important is the enhanced steric interaction of nanoparticles in solution that results in better stabilization of the nanoparticle dispersion, which is due to the large molecular weight of polymers. Polymers can be synthesized comprising different structural subgroups to allow both further functionalization by additional molecules and binding to the nanoparticle surface. Reviews can be found in the literature for a detailed overview on the application of polymers for nanoparticle surface coating [178,179,180,181]. The here-discussed polymers are sketched in Figure 7.

Polyvinylpyrrolidone (PVP) binds to the surface of metal and metal oxide nanoparticles through the carbonyl group of the lactam ring [182]. This can be exploited to coat the surface of Ni nanorods with PVP as dispersion stabilizing surfactant, which has been reported by adding PVP to either a 20 mM sodium hydroxide solution or a sodium hydroxide solution at pH 11.5 [64,165]. The so obtained solution was employed for slowly dissolving the aluminum oxide of the template with the goal that the PVP binds to the nanorods before the template is entirely dissolved. A nanorod dispersion in water was obtained by succeeding washing steps that were assisted by centrifugation and permanent magnets [64,165].

It is reported in the literature that branched polyethyleneimine (PEI) can be used for surface modification of Ni nanorods [166]. To that end, an aqueous solution of branched PEI at a pH of 5.5 was added to an aqueous solution of Ni nanorods and stirred for 1 h. Though this protocol is mainly based on electrostatic binding of branched PEI to the nanorod surface, hydrogen bonding between the amino groups of the polymer and the hydrogen oxide of the native oxide may also be involved in the surface coating process. The advantage of using branched PEI is that it can be modified prior to the nanorod surface coating step by making use of the amino groups of the polymer. This was shown by labeling the branched PEI with a fluorescent organic dye to yield fluorescent nanorods.

An alternative approach to yield polymer coated Ni nanorods was presented by Tripathy et al., who added polyethylene glycol (PEG) to the electrodeposition solution containing the Ni cations (Ni^2+^) [167]. This lead to the formation of coordination bonds between the metal cations and the polymer. By employing this solution for the electrodeposition process into alumina templates, the polymer was deposited at the same time as the nickel. The encapsulation effect of the nickel nanorod by the polymer was due to the positively charged pore walls that lead to the reduction of the Ni cations in the pore center, while the polymer adsorbed to the pore walls. After immersion of the template in a 0.1 M sodium hydroxide solution overnight, nanorod dispersions were obtained.

Finally, Son et al. applied immersion in methanol at 4 °C to bind RGD peptides (a sequence of the three amino acids arginine, glycine, and aspartic acid) to the Ni nanorod surface [132]. Here, for linking to the nanorod, a polyethylene glycol (PEG) spacer comprising an amine and a carboxyl group was applied. First, the PEG was bound by EDC/NHS linker chemistry with its amine end to the peptide (specifically to the carboxyl groups of the aspartic acid), followed by another binding step of the carboxyl end group to the nanorod.

### 3.4. Surface Modification by Histidine

**Figure 8:**
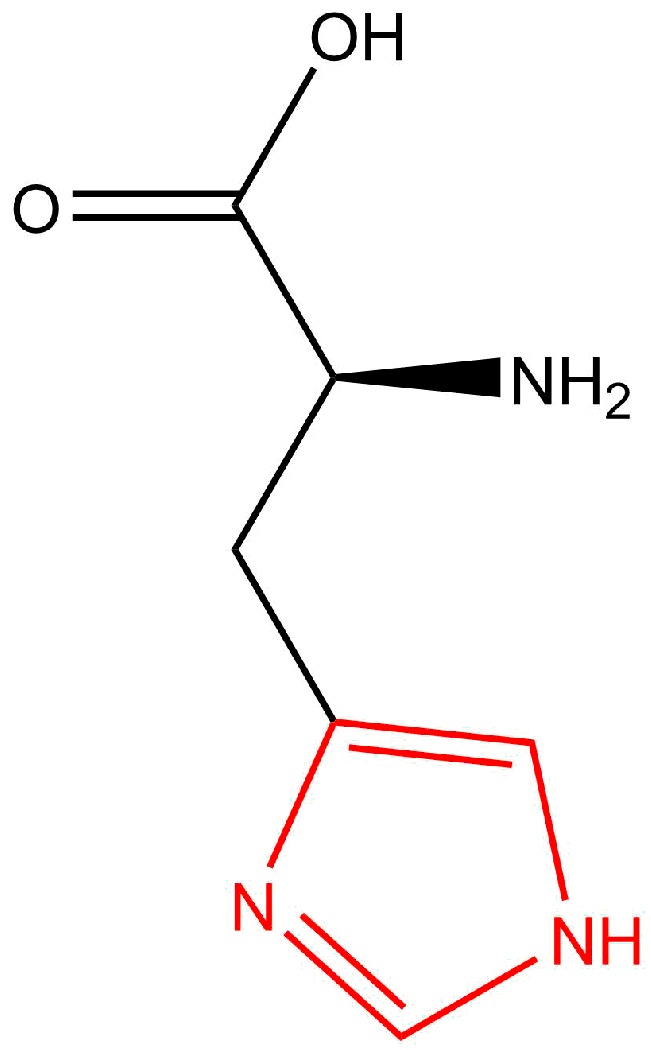
The amino acid histidine with the imidazole group (red) as part of the side chain.

Proteins with histidine amino acid side chains are reported to have a high affinity for nickel, which is also employed for protein purification processes [183,184]. Thus, molecules with a histidine tag can be employed for surface modification of Ni nanorods. The structure of histidine is depicted in Figure 8, with the imidazole group that is a part of the amino acid side chain shown in red.

Choi et al. demonstrated the binding of a His_6_-tagged peptide to the nickel surface of Ni-Au segmented nanorods [129]. After removal of the aluminum oxide template in sodium hydroxide and transfer of the nanorods to phosphate buffered saline solution at a pH of 7.4, the His-tagged peptide was added in an aqueous solution followed by an immersion step for 8 h at 4 °C. Binding of the histidine to the Ni surface was accomplished via the imidazole side chain of the amino acid. Recently, Ho et al. reported the surface modification of Ni-Co compound nanorods by a His_5_-tagged biotin [168]. Here, an array of nanorods that were freestanding on a titanium substrate were immersed together with His_5_-tagged biotin in a phosphate buffered saline solution at a pH of 6.5 for 2 h at 35 °C. This procedure opens up the possibility to further modify the surface by streptavidin, which can be followed by immobilization of another biotinylated molecule as each streptavidin molecule possesses the four binding sites for biotin. Streptavidin labeled by a fluorescent dye was employed by Ho et al. to prove the successful binding of His-tagged biotin to the nanorod surface.

### 3.5. Metal or Metal-Oxide Shell Growth

**Figure 9:**
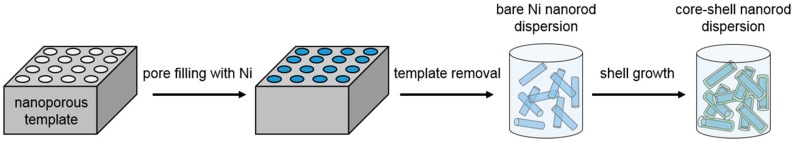
Schematic sketch of the synthesis procedure for core-shell nanorods. First, the pores of a nanoporous template are filled with Ni by electrodeposition. Then, the template is removed so that a dispersion of bare Ni nanorods is obtained. Finally, nanorods in solution are coated by a metal or metal oxide shell.

The overgrowth of the nanorod by an additional metal or metal oxide shell presents another possibility for surface modification with distinct advantages. These are the protection of the core towards any degradation, the increase of the number of established protocols for possible further surface modifications (depending on the materials employed for the shell), and additional physical properties as, for example, localized surface plasmon resonances in the case of gold shells. Several reviews can be found in the literature dealing with the synthesis, the properties and the applications of core-shell nanoparticles [185,186,187]. The synthesis of core-shell nanorods is typically accomplished after the electrodeposition process once the template was removed, which is schematically shown in Figure 9.

The overgrowth of electrodeposited Ni nanorods by a gold shell was accomplished by Jeon et al. employing an electroless gold plating process [169]. To that end, Ni nanorods were first released from the alumina template by dissolving the aluminum oxide in a solution of sodium hydroxide. The dispersed nanorods in solution were then washed and transferred to water. Gold shell synthesis was achieved by immersing the nanorods in an aqueous potassium gold cyanide solution at a temperature of 90 °C. The so-fabricated core-shell nanorods were functionalized by using thiol linkers for binding to the gold shell.

Pondman et al. reported the deposition of a gold shell onto the surface of Ni nanorods that were electrodeposited into polycarbonate membranes [156]. After washing steps and transfer of the nanorods into aqueous solution assisted by centrifugation, the nanorods were coated by a gold layer based on a method presented by Kohli et al. for electroless synthesis of gold nanorods [188]. Here, we briefly describe the procedure, while the details can be found in the original report. First, the nanorods were coated with aminoundecanoic acid, which resulted in the formation of amine groups on top of the nanorod surface [156]. In a subsequent step, tin cations (Sn^2+^) were bound to the amine groups by immersion in a tin chloride solution, followed by oxidation to Sn^4+^ by silver nitrate, which resulted in the formation of a layer of silver atoms on top of the nanorod surface. This silver layer was then replaced by gold atoms via immersion into a commercially available gold solution, which was followed by a continuous growth of a metallic gold layer. Biofunctionalization of the gold layer was then possible by the well-known interaction of gold with thiol groups.

Serrà et al. recently presented the deposition of gold onto electrodeposited Ni nanorods by immersing the nanorods in an aqueous solution of chloroauric acid, which lead to the formation of gold by galvanic displacement [170]. A subsequent functionalization step was carried out by binding a polyethylene glycol polymer comprising a thiol group to the nanorod surface.

Nanoparticles can also be coated by a shell of silica (silicon dioxide), which is a biocompatible material that protects the core and allows for further functionalization of the surface, e.g., by silane groups. A widely employed method for the growth of a silica layer is the so called Stöber process, where the silica shell is grown by condensation of the precursor tetraethoxysilane (TEOS) in a solution of ethanol and ammonia [189]. Pinheiro et al. employed the silica shell growth for electrodeposited Ni nanorods once they were released from the alumina template and transferred to ethanol [123]. Specifically, they employed co-condensation of TEOS and siloxydithiocarbamate to generate dithiocarbamate groups on the silica shell. Dithiocarbamate groups have a high binding affinity for heavy metal ions. Here, the siloxydithiocarbamate precursor was prepared prior to the silica shell growth by linking a dithiocarbamate group to (3-aminopropyl)triethoxysilane.

A further possibility to overcoat a nanorod surface by a silica shell was presented by Graf et al. [190]. Here, they coated a range of different nanoparticles by employing PVP as a sub-layer for subsequent silica coating in a Stöber growth process. This has been employed for electrodeposited Ni nanorods that were released form the template in a sodium hydroxide solution containing PVP at a pH of 11.5 [171]. After some washing steps and a transfer to water, the nanorods were dispersed in an aqueous solution of 2-propanol and ammonia (25%), and then TEOS was added to induce the silica shell growth. After additional washing steps assisted by centrifugation and magnetic separation, silica coated Ni nanorod dispersions in water were obtained.

## 4. Conclusions and Outlook

In this review, we focused on Ni nanorods that have been fabricated by electrodeposition into porous membranes. Such ferromagnetic nanorods offer the advantage that they already intrinsically possess different functionalities. Specifically, their elongated form leads to magnetic shape anisotropy that fixes their magnetic moment in the direction of the long nanorod axis. At the same time, the rod-shaped form also results in an optical anisotropy. Consequently, it is possible to examine the actual nanorod orientation in a solution either by magnetic or optical sensing methods. Moreover, the orientation of nanorods can be manipulated by external magnetic fields, so that a wide field of applications can be realized.

We presented the extensive scope of applications of these nanorods, which span from electronics and data storage to biosensing and to cell biology, including both fundamental cell biology and cancer treatment. The second main part of the review focused on nanorod surface modification strategies to allow for fabrication of customized nanorods that can be applied for a specific application. A suitable surface modification of nanorods is crucial for their application in complex measurement settings. Thus, it will play an important role for a future broadening of the range of Ni nanorod applications. Regarding this broadening of the applicability, several different possibilities can be easily envisaged. Firstly, segmented nanorods with different materials to increase the functionality of the bare nanorods itself present a promising future direction that can open up new fields of application. Secondly, Ni nanorods could be employed for all imaging and detection techniques that rely on either a magnetic or an optical detection. Examples are magnetic particle imaging, photoacoustic imaging or optical coherence tomography, next to the already presented magnetic resonance imaging and surface-enhanced Raman spectroscopy. This is of special interest when biofunctionalized nanorods can be employed. Alternatively, one might expect that Ni nanorods can be employed for any kind of tracking measurements, as nanorods can be sensitively detected by their magnetic and optical characteristics. An example would be to track the path of a sample fluid within a complex setting. Moreover, Ni nanorods can be employed for measurements at a single cell level to examine processes inside living cells. Thirdly, in order to broaden the applicability of Ni nanorods, the surface modification can be improved. This accounts for advanced protocols for the binding of antibodies and peptides, which will be an improvement for all sensing and biosensing applications. Furthermore, a surface modification that results in nanorods that withstand harsh environmental conditions can trigger the development of new applications. Finally, the synthesis of more complex polymers that already possess an intrinsic multifunctionality can be an important future step. In the following final paragraphs, we will discuss alternative possible surface modification approaches for which, to our knowledge, no protocol for the here discussed Ni nanorods can be found in the literature. This, for sure, is an incomplete list, but it might be a motivation that triggers new developments.

As discussed above, carboxylic acid groups can be employed for Ni nanorod surface coating as a result of their affinity to nickel oxide. Similarly, all kinds of fatty acids could be employed for surface modification. Moreover, polyacrylic acid (PAA) with a carboxyl group in every monomer unit is a promising candidate for Ni nanorod surface modification. It offers the additional advantage that some of the carboxyl groups of the polymer can be used to modify the polymer by further functional molecules.

Alternatively, alkylphosphonic acids were reported to bind to native nickel oxide surfaces [191]. Here, the presented immersion method can be a promising approach to attach alkylphosphonic acids to electrodeposited Ni nanorods. These molecules, which comprise a phosphonic group, can also be modified with another functional group at the opposite end of the alkyl chain. For example, 16-phosphonohexadecanoic acid that possesses a carboxyl group. This additional functionality could then be employed to add another molecule for further nanorod surface modification.

Another simple approach for a surface modification was based on the use of commercially available spherical Ni nanoparticles. These were coated by the also commercially available Brij 76 ligand that has poly(ethylene glycol) octadecyl ether as main ingredient, which allows for a transfer from aqueous to organic solutions [192]. This could be exploited for all applications that do not necessarily rely on water-based nanorod dispersions.

Regarding the synthesis of metal or metal-oxide shells on top of the nanorod surface, various alternative materials could be employed. For example, it has been reported that Ni nanorods that were synthesized by a template-free method were coated by tellurium and also by zinc oxide shells [193]. This resulted in hybrid nanorods with magnetic and semiconducting properties, which can also be a very interesting approach for template-synthesized nanorods. Therefore, an additional functionality could already be realized for the bare nanorod prior to any surface modification.

Finally, Solanki et al. immobilized protein A on the surface of nickel oxide nanowires [194]. Though this method was based on an electrostatic interaction between the nickel oxide and protein A, the authors were able to demonstrate its application for biosensing. By making use of the well-known affinity of the Fc fragment of antibodies to protein A, they were able to further modify the nanowire surface by antibodies. In principle, it could be possible to expand this approach to electrodeposited Ni nanorods, but also to alternative established protein-antibody interactions. These approaches could be based, for example, on protein G or on protein A/G.

In summary, it can be concluded that the already existing wide range of applications of Ni nanorods will further grow in the future. This will be a result of the multifunctionality of these kinds of nanoparticles. The development of new advanced surface modification procedures will further boost the application of Ni nanorods and will open new fields of applications. Thus, one can be very curious to see the advances of this scientific field in the coming years.

## Figures and Tables

**Table 1:** Summary of Ni nanorod applications.

Field of Application	Comments	References
Rheological fluid properties	Hydrogels	[89,90,91,92,93]
	Micellar solutions	[94,95]
	Interfacial shear rheology	[96,97]
Data storage	Multilayered Ni-Cu nanorods	[98]
Electronics	Microwave electronics	[99,100,101,102,103,104]
Catalysis	Catalysis of methanol	[51,105]
	Oxygen reduction	[106]
Optical phenomena	Localized surface plasmon resonance	[107,108,109]
	Surface-enhanced Raman scattering	[110,111,112]
	Liquid crystal technology	[113,114]
Sensing and biosensing	Carbohydrates	[115,116,117,118,119,120]
	Proteins	[121]
	Magnetic resonance imaging	[122]
	Heavy metal ions	[123]
Cell biology	Internalized nanorods with external agitation	[124,125,126]
	Release/presentation of target molecules	[127,128,129,130,131,132]
	Cell guidance/cell growth guidance	[133,134,135,136]
	Cell separation	[137,138,139,140]
	Inducing cell death	[141,142,143,144]

**Table 2:** Summary of surface modification strategies.

Surface Modification	Molecule/Shell Material	References
Small molecules based on carboxylic acid groups	Hematoporphyrin	[160,161]
	Pimelic acid	[140]
	Palmitic acid	[162]
	AEDP ^1^	[128]
	Folic acid	[130]
Small molecules based on silane groups	APTES ^2^	[163]
	APTMS ^3^	[164]
Polymers	PVP ^4^	[64,165]
	Branched PEI ^5^	[166]
	PEG ^6^	[167]
	RGD peptide ^7^	[132]
Histidine	Histidine	[129,168]
Metal/metal oxide shell growth	Gold	[156,169,170]
	Silica	[123,171]

^1^ 3-[(2-aminoethyl)dithio] propionic acid; ^2^ (3-aminopropyl)triethoxysilane; ^3^ (3-aminopropyl) trimethoxysilane; ^4^ polyvinylpyrrolidon; ^5^ polyethyleneimine; ^6^ polyethylene glycol; ^7^ sequence of the amino acids arginine, glycine, and aspartic acid.
